# The Pandemic, Infodemic, and People’s Resilience in India: Viewpoint

**DOI:** 10.2196/31645

**Published:** 2021-12-08

**Authors:** Shabbir Syed Abdul, Meghna Ramaswamy, Luis Fernandez-Luque, Oommen John, Thejkiran Pitti, Babita Parashar

**Affiliations:** 1 Graduate Institute of Biomedical Informatics Taipei Medical University Taipei Taiwan; 2 School of Gerontology Health Management Taipei Medical University Taipei Taiwan; 3 International Office University of Saskatchewan Saskatoon, SK Canada; 4 Adhera Health Inc Palo Alto, CA United States; 5 George Institute for Global Health University of New South Wales New Delhi India; 6 Manav Rachna University Faridabad India

**Keywords:** pandemic, COVID-19, India, digital health, infodemics, Sustainable Development Goals, SDGs

## Abstract

The COVID-19 pandemic, caused by severe acute respiratory syndrome coronavirus 2 (SARS-CoV-2), has caused widespread fear and stress. The pandemic has affected everyone, everywhere, and created systemic inequities, leaving no one behind. In India alone, more than 34,094,373 confirmed COVID-19 cases and 452,454 related deaths have been reported as of October 19, 2021. Around May 2021, the daily number of new COVID-19 cases crossed the 400,000 mark, seriously hampering the health care system. Despite the devastating situation, the public response was seen through their efforts to come forward with innovative ideas for potential ways to combat the pandemic, for instance, dealing with the shortage of oxygen cylinders and hospital bed availability. With increasing COVID-19 vaccination rates since September 2021, along with the diminishing number of daily new cases, the country is conducting preventive and preparatory measures for the third wave. In this article, we propose the pivotal role of public participation and digital solutions to re-establish our society and describe how Sustainable Development Goals (SDGs) can support eHealth initiatives and mitigate infodemics to tackle a postpandemic situation. This viewpoint reflects that the COVID-19 pandemic has featured a need to bring together research findings across disciplines, build greater coherence within the field, and be a driving force for multi-sectoral, cross-disciplinary collaboration. The article also highlights the various needs to develop digital solutions that can be applied to pandemic situations and be reprocessed to focus on other SDGs. Promoting the use of digital health care solutions to implement preventive measures can be enhanced by public empowerment and engagement. Wearable technologies can be efficiently used for remote monitoring or home-based care for patients with chronic conditions. Furthermore, the development and implementation of informational tools can aid the improvement of well-being and dissolve panic-ridden behaviors contributing toward infodemics. Thus, a call to action for an observatory of digital health initiatives on COVID-19 is required to share the main conclusions and lessons learned in terms of resilience, crisis mitigation, and preparedness.

## Introduction

The rapid spread of COVID-19, caused by severe acute respiratory syndrome coronavirus 2 (SARS-CoV-2), has resulted in extensive panic among the public. The COVID-19 pandemic has impaired social values as well as the economy of the country, thereby creating systemic inequities [1]. In a highly populous country like India, the pandemic has pulled down the economic progress attained in recent years by people belonging to lower and middle socioeconomic classes, and it has pushed 230 million people into poverty [2]. In India alone, there were more than 34,094,373 confirmed COVID-19 cases and 452,454 related deaths reported as of October 19, 2021 [3]. With most Indian cities reporting COVID-19 cases and a government restriction on operating hours for businesses, there has been a large exodus of workers from cities to rural areas. This raised a unique challenge for the health system, as rural areas lacked health infrastructure such as medical supplies and equipment required for testing and providing essential health care to people (see Multimedia Appendix 1). The dire snapshot of the COVID-19 crisis in India reflects underinvestment in both its health care and public health system. Moreover, misinformation circulating in social media has driven panic at a pace and scale never experienced before. A study surveyed panic levels, ranging from 0 (minimum) to 5 (maximum), among 1075 social media users, wherein higher panic levels were reported among Indian users [4].

Following the first case of COVID-19 reported in India, the government took a bold step to lock down the country of 1.3 billion people [5]. India was proactive in curbing the COVID-19 pandemic by taking steps to strengthen its health care system and infrastructure and by manufacturing personal protective equipment (PPE). A national task force was established by the Indian Council of Medical Research to initiate research studies and identify priorities for clinical research, epidemiology, surveillance, diagnostics, biomarkers, vaccines, and drug development [6]. As a result, India reported one of the lowest rates of COVID-19–related mortality in the early stages of the pandemic. Nonenforcement of policies by the Indian government to ensure public adherence to face masks, sanitation, hygiene, and social distancing likely caused silent widespread transmission of COVID-19 [7].

**Figure 1:**
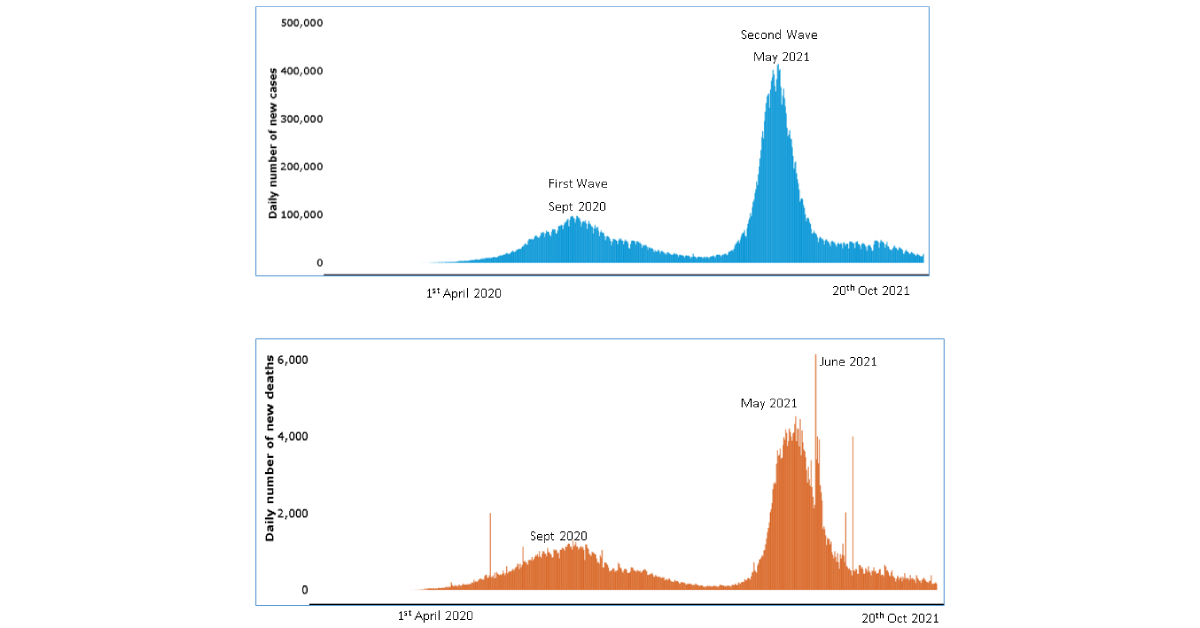
The trend of daily new cases and deaths in India from April 2020 to October 2021; adapted from the World Health Organization COVID-19 Explorer [8].

The second wave of COVID-19 had proven to be rampant and virulent (Figure 1 [8]). The new “double mutant variant” of coronavirus had been detected, which was considered a variant of concern due to its immune escape properties, and it was known to have high infectivity and transmission rates. Despite these variants of concern, other possible factors for the surge in cases likely include noncompliance of COVID-19–appropriate behavior by the citizens and the widespread reopening of economic activity. In addition, general elections in multiple Indian states and religious mass gatherings, such as the “Kumbh Mela” (from April 1 to 17, 2021), were considered as super-spreader events, as they resulted in thousands of COVID-19–positive cases (see Multimedia Appendix 1). As pilgrims continued their travels and returned home, they further spread the infection in cities across India.

One major challenge related to COVID-19 in India was the apparent lack of data access and availability for analysis or data modeling from the Indian Council of Medical Research [9,10]. The delayed access and the lack of testing and sequencing capacities led to the sequencing of less than 1% of total positive samples, compared to 4% in the United States and 8% in the United Kingdom [9]. Despite these delays and rising concerns, the general public carried out various measures to mitigate the infection spread by using emerging technologies and social media strategies. Through this viewpoint, we propose the pivotal role of digital solutions and public participation to re-establish our society and describe how Sustainable Development Goals (SDGs) can support eHealth initiatives. In the sections below, we describe the situation of overwhelming health systems, community resilience initiatives and their implications in health care delivery, and finally, how digital health solutions can help achieve SDGs and mitigate pandemics.

## Overwhelming the Capacity of Health Systems

Despite the slow and gradual increase in the number of COVID-19 cases since January 2021, it was only on April 2, 2021, that the government raised alarm, labeling the situation as grim and serious. Although the evidence from other geographical territories showed that a rapid increase in COVID-19 cases could seriously disrupt health delivery systems, create stress in the health workforce, limit access to hospital services, and increase mortality, limited efforts were made to address this surge capacity. In May 2020, an analysis by National Institution for Transforming India (NITI) Aayog—India’s nodal policy planning agency—identified the severe dearth of medical equipment, such as testing kits, PPE, masks, and ventilators. The agency also noted the long-running shortage of emergency health care and lack of professionals, with the ratio of doctors to patients recorded as 1:1445, that of hospital beds to people recorded as 0.7:1000, and that of ventilator to population recorded as 40,000 to 1.3 billion (1:130,000) [11]. Efforts to address these gaps comprehensively have been inadequate.

## Community Resilience Initiatives and Their implications for Health Services Delivery Models

In the last few years, there has been an exponential growth in the use of digital technologies in the Indian population. Furthermore, there has been a rapid development of telehealth services through the web-based registration system in India that leverages the expanding health information technology infrastructure [12-15]. Moreover, India is one of the powerhouses for the development of mobile and web-based solutions. As such, one should not be surprised that many digital solutions are emerging, such as the development of groups and websites to assist people in finding crematoriums amidst the ongoing devastating crisis or locating hospitals with available beds [16,17]. Apart from the government, community-based initiatives such as the involvement of religious organizations, welfare groups such as the Rotary Club and Lions Club, as well as individuals and social influencers were at the frontline to spread awareness and provide support [18]. Digital reach has further empowered the urban community groups to catalyze their initiative from COVID-19 awareness to mitigation. All these initiatives had an impact on countless families and were often led by the community. This impact included ensuring proper information sharing and health communication, training of primary health care workers in identifying and deflating misinformation and providing simple and relevant sources of updated information [19,20]. These are prime examples of citizen science and participatory health [21].

Social media emerged as a glimmer of hope amid the ongoing COVID-19 crisis. With hospitals struggling to maintain enough medical supplies and preventing shortage of oxygen, several people have resorted to sending out SOS calls on social media. Most people publishing such posts on social media platforms have been seeking beds, oxygen, and convalescent plasma. Hospitals across India have also been regularly using social media hashtags, such as #Covid19IndiaHelp, #SOSDelhi, and #helpcovidindia, on Twitter to seek urgent help, specifically to circumvent oxygen shortages [22]. Several web-based resources have been a source of crucial information, for example, a data science platform was used to collate various resources, ranging from oxygen to intensive care unit (ICU) beds and essential medicines from different places across India [12,16,17]. Many of these resources provide a comprehensive dashboard for COVID-19 resources in Indian cities. Mobile apps were able to provide information on oxygen cylinders, ICU beds, medicines, and plasma availability. Twitter India launched a *COVID-19 Resources* page featuring SOS calls and tweets that offer help to patients who require services, such as ambulance, oxygen, medicines, and ICU beds. Volunteer-led platforms such as Project StepOne offered tele-triage and teleconsultations for individuals with COVID-19 symptoms and provided self-management support at home through remote monitoring. This platform has over 7000 impaneled doctors and is partnering with 16 state governments to address COVID-19 management through telemedicine solutions [23,24].

## Digital Health and SDGs to Mitigate Infodemics

Misinformation is its own pandemic [25]. The uncertainties related to diagnosis and treatment of COVID-19 have led to a significant growth of health misinformation, transforming an infrastructure of health promotion into that of health conspiracy. Misinformation does not stop at national borders and requires the development and coordination of initiatives, with partners to promote and ensure healthy lives and well-being for people of all age groups. The COVID-19 pandemic has highlighted a need to bring together findings across disciplines, build greater coherence within the field, and serve as a driving force for multi-sectoral, cross-disciplinary collaboration. In the first few months of the COVID-19 pandemic, about 2300 reports on COVID-19–related misinformation were published in 25 different languages across 87 countries. Following the spread of misinformation, 5876 hospitalizations and 800 deaths were reported [26]. The United Nations SDGs are extremely relevant, as they help us understand the broad impacts of the COVID-19 crisis through an economic, social, and environmental lens and play an important role in ensuring that one crisis does not fuel the development of another. Disease and poverty may interact with each other, especially considering that over 736 million people in the world live in extreme poverty and are unduly affected by ill health, thus impacting SDG1 (no poverty) [20]. Furthermore, the COVID-19 crisis is expected to generate increasing food insecurity, especially among low-income groups. This can affect SDG 2 (zero hunger) and surge the need for food sources and public nutrition provision [20]. Published studies indicate that the SDG for good health and well-being (SDG3) will be difficult to achieve, as the COVID-19 pandemic has caused India to delve into poverty and inequality [27].

Disinformation works against the purpose of education and learning. Collective mobilization of knowledge to promote quality education (SDG4) and lifelong learning provides the necessary tools to fight the tsunami of infodemic exacerbated by the COVID-19 pandemic through teaching (at the individual level) strategies to spot misinformation, verify the source of information, and educate the public about research bias. The transformative effects of digital knowledge, literacy, and skills can enable users to understand ethics and human rights to defend against the fabrication and dissemination of disinformation. Although this may help individuals discern authentic information, it may also undermine their trust in science and lead to disinterest [28].

According to reports, interventions supported via SDG4 may improve learning strategies to identify misinformation; however, they may not necessarily reduce sharing of misinformation [29]. Evidence from a 2020 study suggests that information overload and trust in information on the internet are strong predictors of unverified information sharing [30]. The path forward toward media literacy will need to include educational institutions adopting an evidence-based media literacy curriculum to enable individuals to discern fact from opinion. This has been practiced by many educational institutions around the world. Training provided by WhatsApp and the National Association of Software and Service Companies through in-person events in India and social media posts would be critical to identify misinformation [28]. Educational campaigns and the development of a toolkit to quarantine misinformation could potentially have long-lasting effects on the frequency and effectiveness of media accuracy in social contexts. Furthermore, apps could play a more proactive role by filtering content and increasing restrictions of what information can be freely forwarded. Thus, social media apps do act as a source of motivation for information propagation. In this context, Alvin Toffler rightly said that, in this information and communication technology era, the illiterate will not be those who cannot read and write, but those who cannot distinguish between reliable and misleading information available online [31].

Digital health ecosystems have the potential to fulfill the objectives of SDGs. For example, digital technologies can prevent digital isolation, boost connectivity, and provide access to tools and information, which can provide insightful information on populations to achieve health objectives. Digital health technologies have been at the forefront of the COVID-19 pandemic and have caused a shift toward telehealth (eg, virtual visits, care, and e-prescriptions), mobile apps (patient monitoring), and instant messaging applications (risk assessment and screening, triage, etc) [32-34].

Innovative technologies and smartphone apps can help manage the prevention of disease and treatment regimens. Although the benefits of digital health solutions lean toward SDG3, the connection between other SDGs may not be so direct and requires further exploration. These solutions call for a trained workforce, good governance, funding, and an interdisciplinary and intersectoral approach, bringing together all the main actors in the digital health ecosystem—governments, international organizations, health service institutions, academia, research centers, and the public and private industries [35].

The use of mobile apps has enabled health agencies to remotely provide data to the government on the number of cases, symptoms, and prevention measures during the early stages of COVID-19. For example, a World Health Organization (WHO) Health Alert delivers COVID-19 facts to billions via the WhatsApp mobile app [36]. Although apps using global positioning system (GPS) coordinates raise ethical questions on data privacy, some argue that these are essential to identify COVID-19 hotspots and install strong isolation and quarantine measures in certain locations [37]. The pandemic has demonstrated the usefulness of incorporating digital health solutions into our national health care systems. India launched a mobile app Aarogya Setu for exposure notification and contact tracing—it has been promoted as a digital tool to protect people from COVID-19. Despite being the world’s most downloaded mobile app [38], lack of integration into health systems for effective public health responses has limited its usefulness in the pandemic response [39]. Although there is limited data on the acceptance of these tools, these reports highlight the need for greater participatory research and concerted action toward its holistic assessment and implementation. Taiwan has showcased how technologies can assist in the control of the COVID-19 pandemic. Taiwan has extensively used information and communication technologies and big data analytics [40]. Moreover, proactive public participation to using face masks and hand sanitizer has led to unintended benefits, such as reducing the risk of not only COVID-19 but also other infectious diseases [41].

## Conclusions

The grim COVID-19 situation in India has highlighted the need for better coherence, investment, and public participation, in order to minimize the negative impacts of the pandemic. Although this pandemic has considerably hampered the health care system, it also has given us opportunities to re-establish and restructure its functioning. This is the right time to reorganize the system with new initiatives, such as insurance to cover home care solutions, new policy developments for technology usage, among others. Digital solutions can mitigate infodemics and plays a key role in re-establishing our society through the lens of SDGs. This viewpoint highlights the need to develop and apply digital solutions to pandemic situations and further reprocess to focus on SDGs. Proactive development of educational tools can promote well-being and help dissuade panic-ridden behaviors that lead to infodemics. Public empowerment and engagement are key to promote the use of digital health care solutions for implementing preventive measures. Wearable technologies can be efficiently used for remote monitoring of patients with chronic conditions. Furthermore, the development and implementation of informational tools can aid to improve well-being and dissipate panic-ridden behaviors contributing to infodemics. Thus, a call to action for an observatory of digital health initiatives on COVID-19 is required to share key findings and lessons learned in terms of resilience, crisis mitigation, and preparedness.

## Appendix

Multimedia Appendix 1 Experiences from various parts of India demonstrating panic and resilience among the public during the COVID-19 pandemic.

## Abbreviations

GPS: global positioning system
ICU: intensive care unit
NITI: National Institution for Transforming India
PPE: personal protective equipment
SDG: Sustainable Development Goal
WHO: World Health Organization
