# Supplementary material for: The Pandemic, Infodemic, and People’s Resilience in India: Viewpoint
Source: JMIR Public Health Surveill. 2021 Dec 8;7(12):e31645. doi: 10.2196/31645 (PMC8658220; doi:10.2196/31645)
Supplement: Multimedia Appendix 1 [file publichealth_v7i12e31645_app1.pdf]

# Pandemic, Panic and Peoples Resilience - Experience from India

# Election campaign in Assam

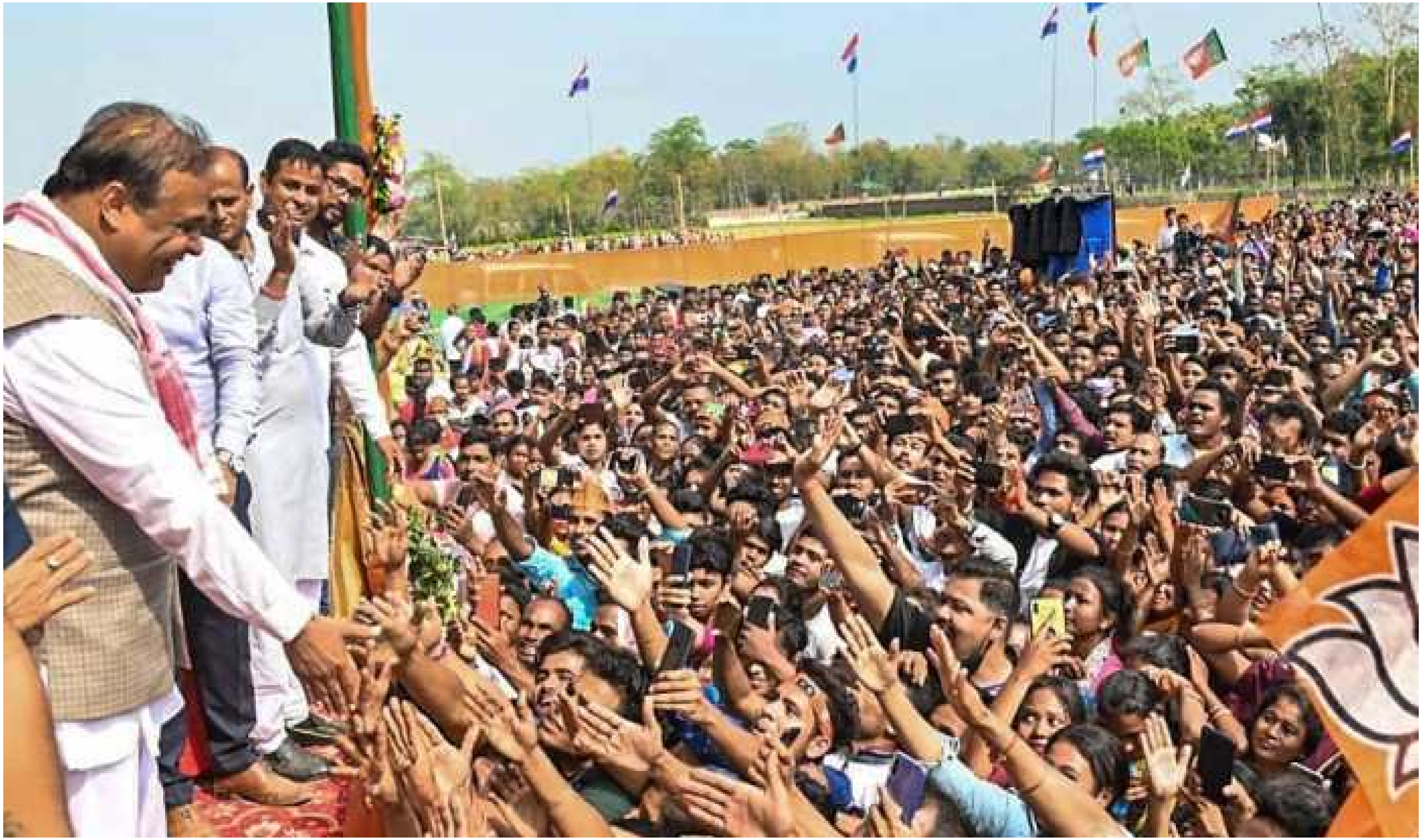

An election campaign rally at Guwahati in Assam (North-eastern part of India) during Assembly election 2021. Source: Zee News; Dated: April 4, 2021

# Election campaign in Assam

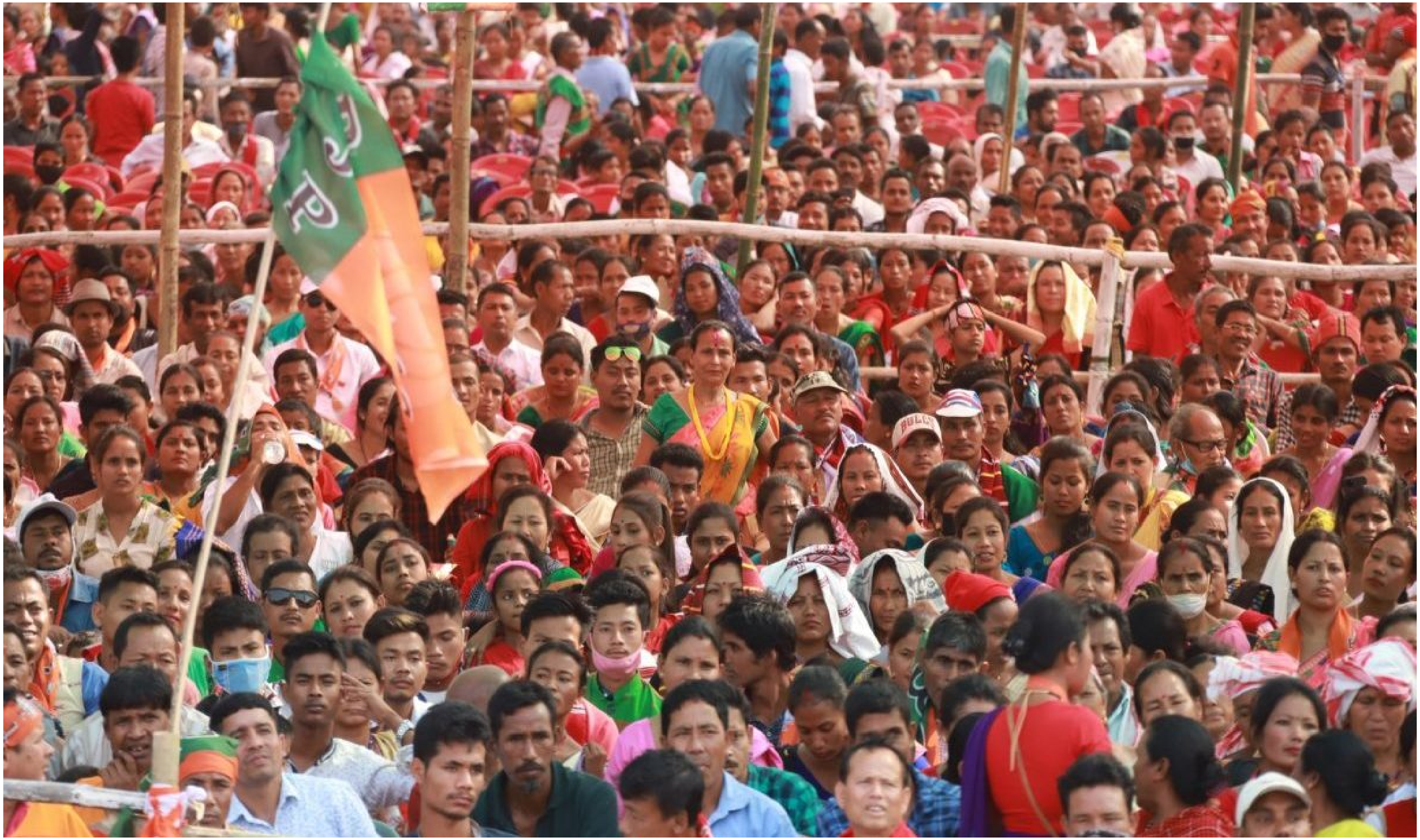

An election campaign at Majuli in Assam (North-eastern part of India) during Assembly election 2021. Source: The Print; Dated: March 24, 2021

# Election campaign in West Bengal

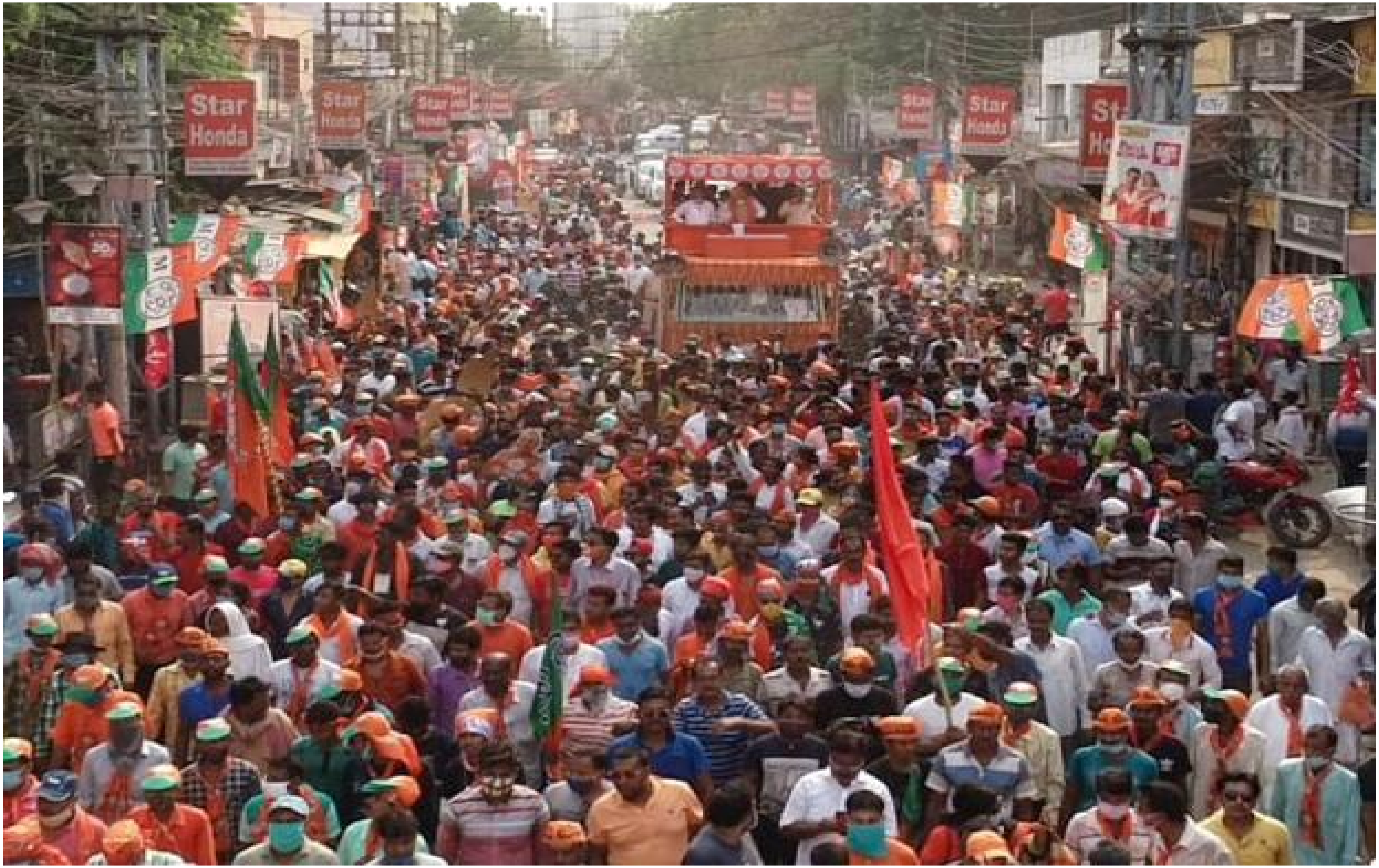


A road show held at election campaign in West Bengal (Eastern part of India) during Assembly election 2021. Source: Business Standard; Dated: April 16, 2021

# Election campaign in West Bengal

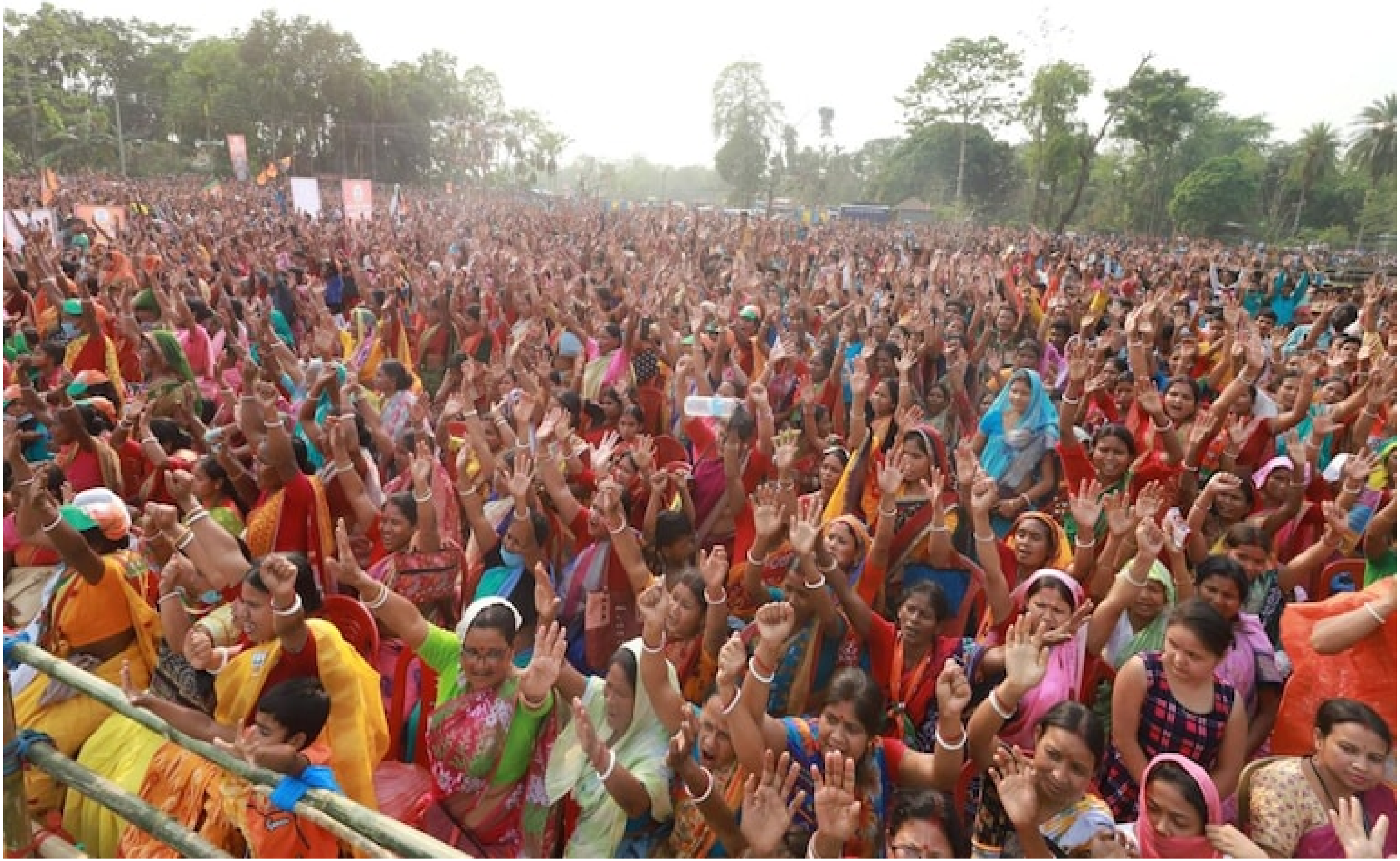

An election campaign in West Bengal (Eastern part of India) during Assembly election 2021. Source: India Today; Dated: April 13, 2021

# Election campaign in Tamil Nadu

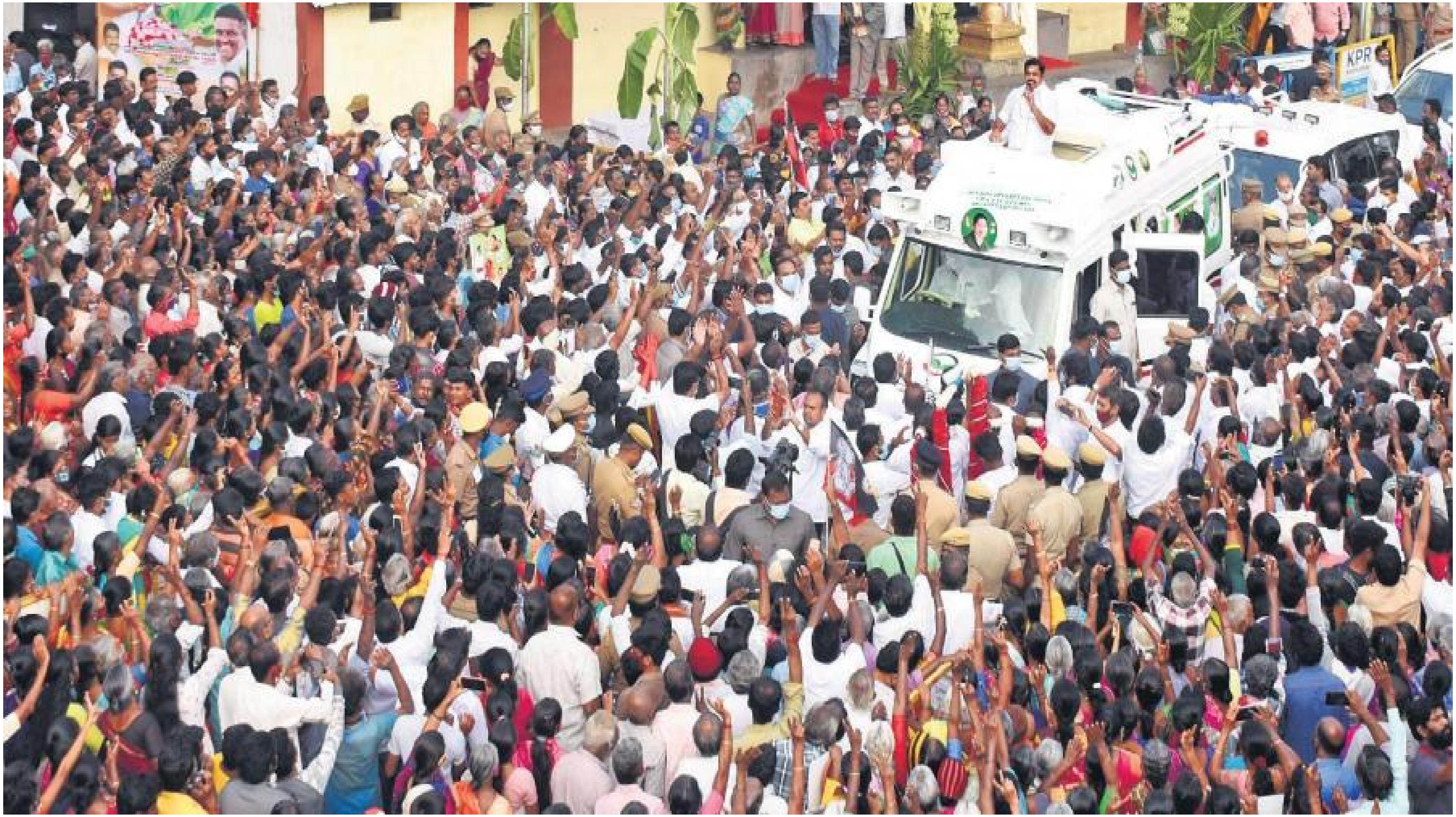


Addressing a crowd during a road rally in election campaign at Puliyakulam in Comibatore, Tamil Nadu (Southern part of India). Source: The New Indian Express; Dated: March 22, 2021

# Kumbh Mela procession in Haridwar

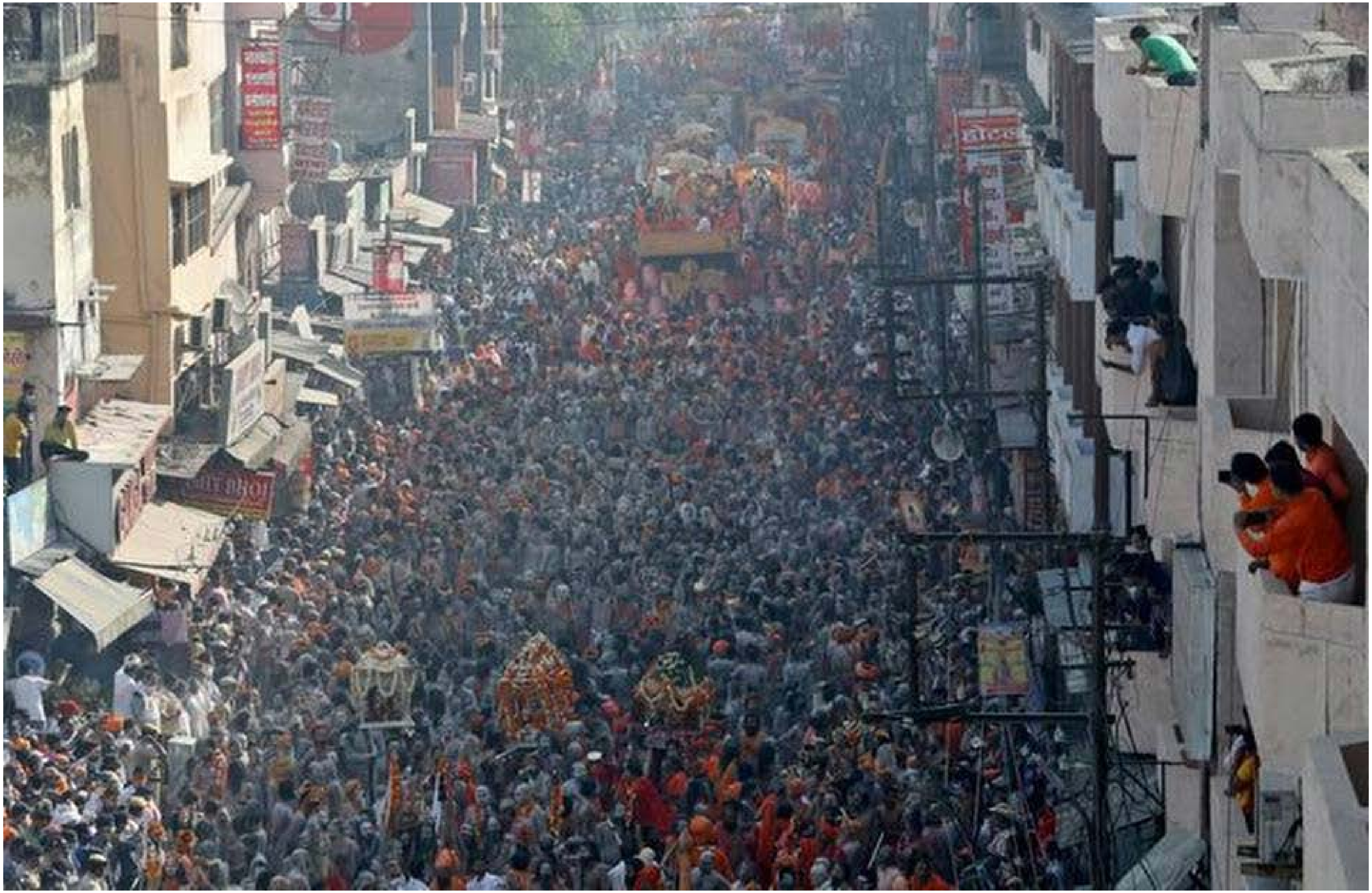

Procession to take a dip in the river Ganges during Shahi Shah at Kumbh Mela in Haridwar (Northern part of India). Source: The Hindu; Dated: April 14, 2021

# Kumbh Mela Holy dip at the Ganges

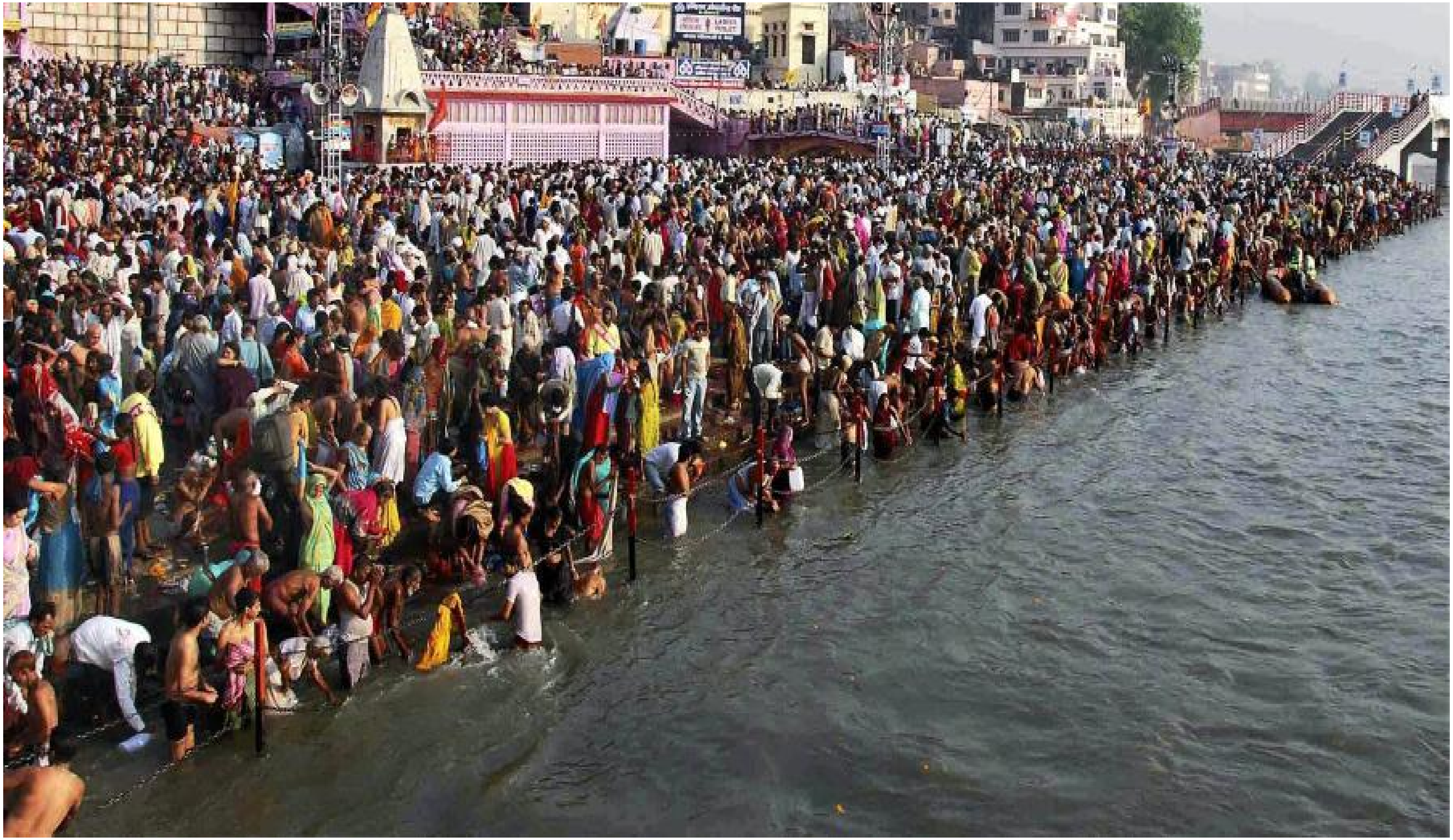

Taking a holy dip in the river Ganges during Shahi Shah at Kumbh Mela in Haridwar. Source: The New Indian Express; Dated: April 15, 2021

# Religious procession in Gujarat

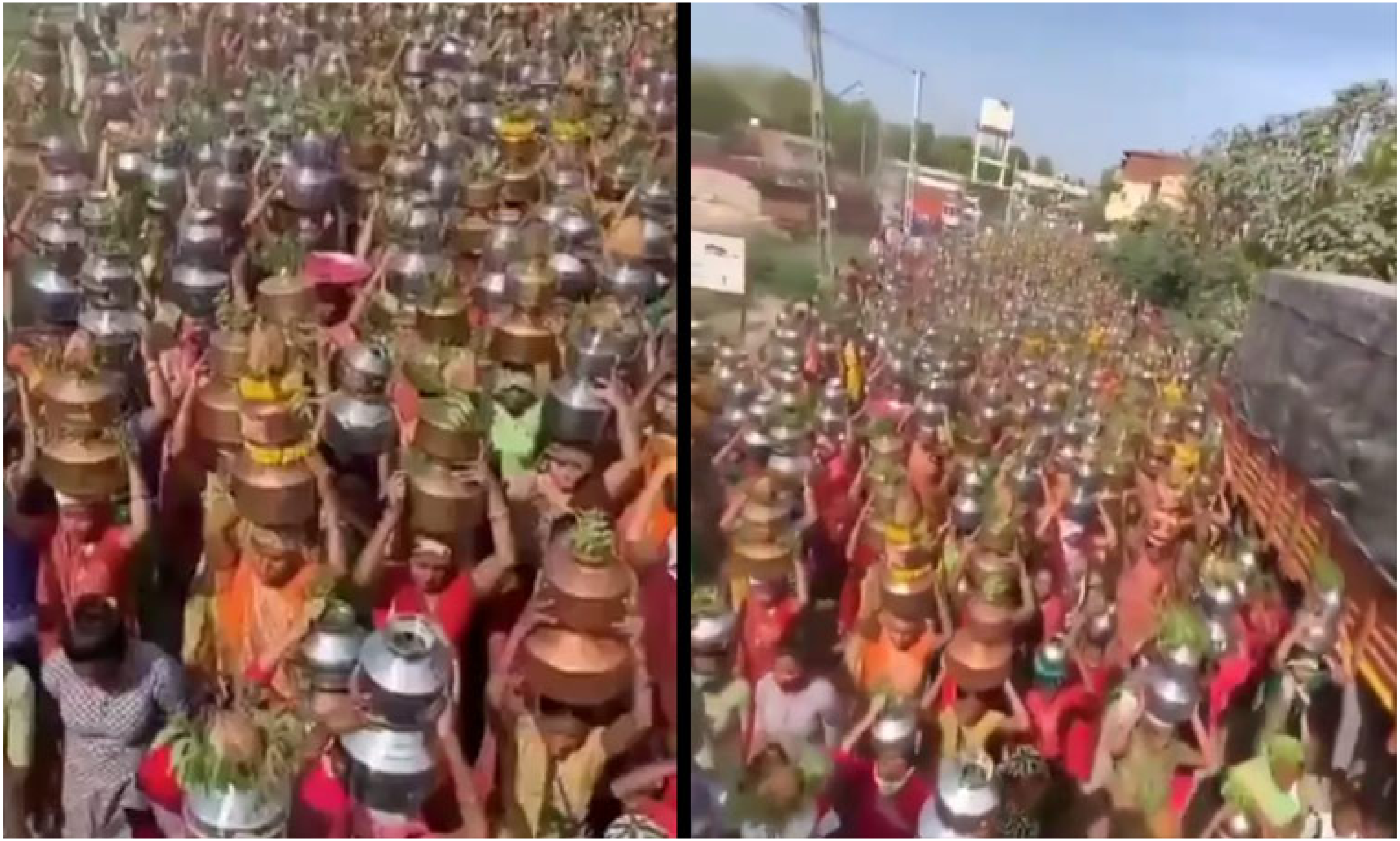

Large number of women turned up at a religious event organized in Navapura village in Ahmedabad district, Gujarat (Western part of India) to eradicate coronavirus. Source: The Week; Dated: May 5, 2021

# Massive exodus of migrant workers

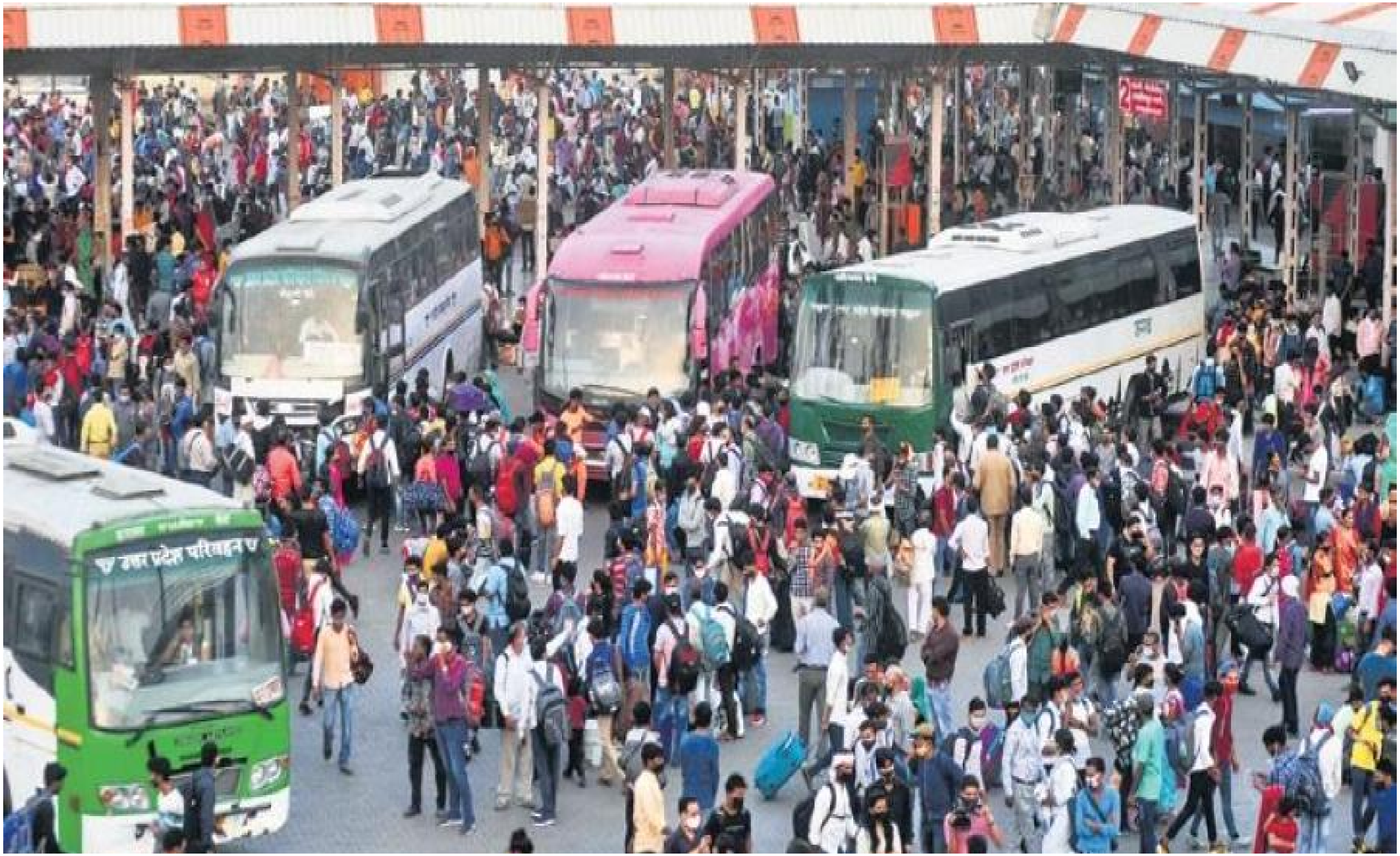

Massive exodus of migrant workers wait to catch a bus at Kaushambi bus stand, Anand Vihar in East Delhi after lockdown announced in Delhi (Northern part of India). Source: The New Indian Express; Dated: April 20, 2021

# Two to a bed in Delhi hospital

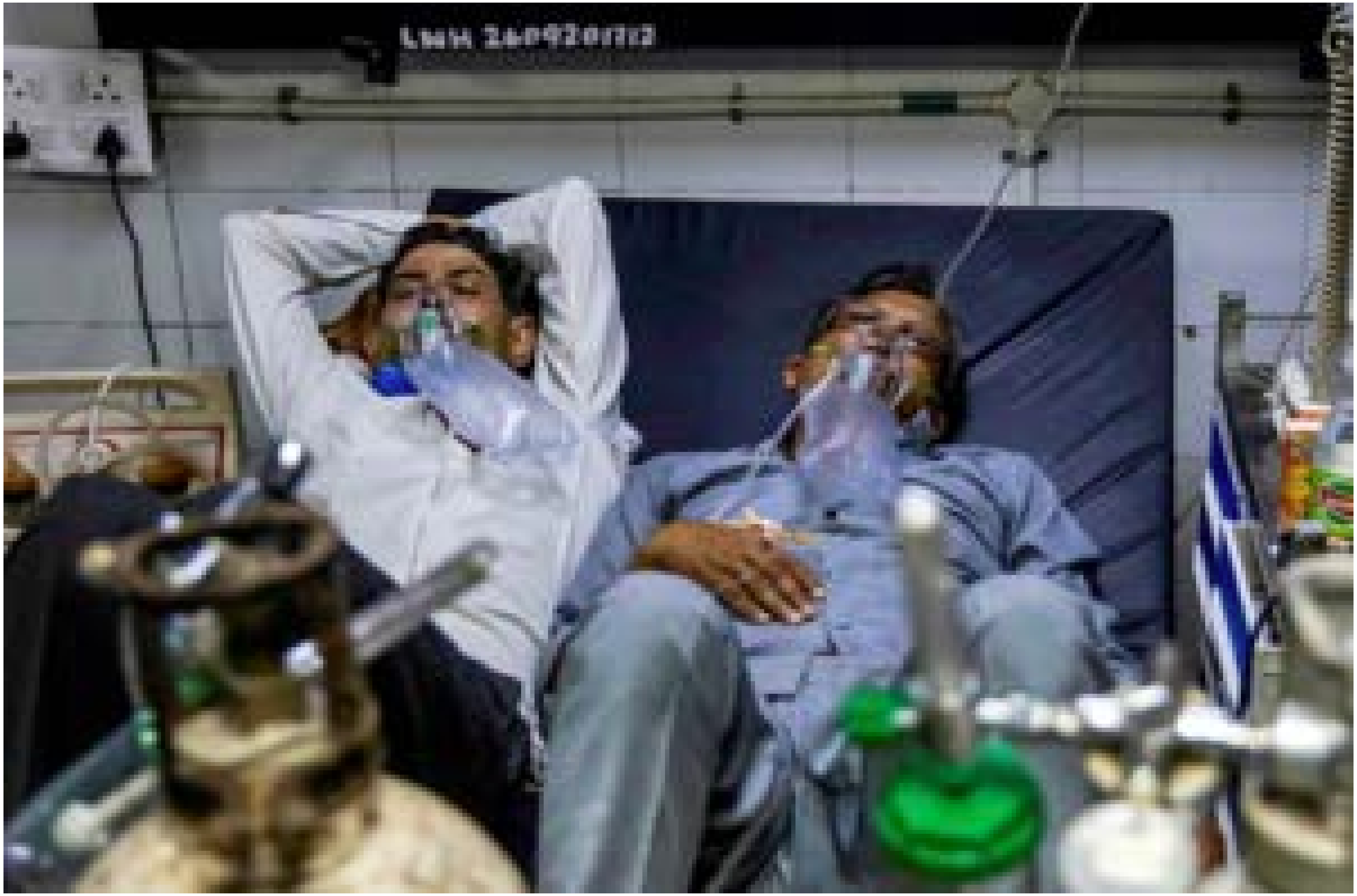

At Lok Nayak Jai Prakash Narayan Hospital  in New Delhi, one of India's largest COVID-only facilities with more than 1,500 beds, a stream of ambulances ferried patients to the overflowing casualty ward. Source: The Economic Times; Dated: April 15, 2021

# Long wait for beds with oxygen supply

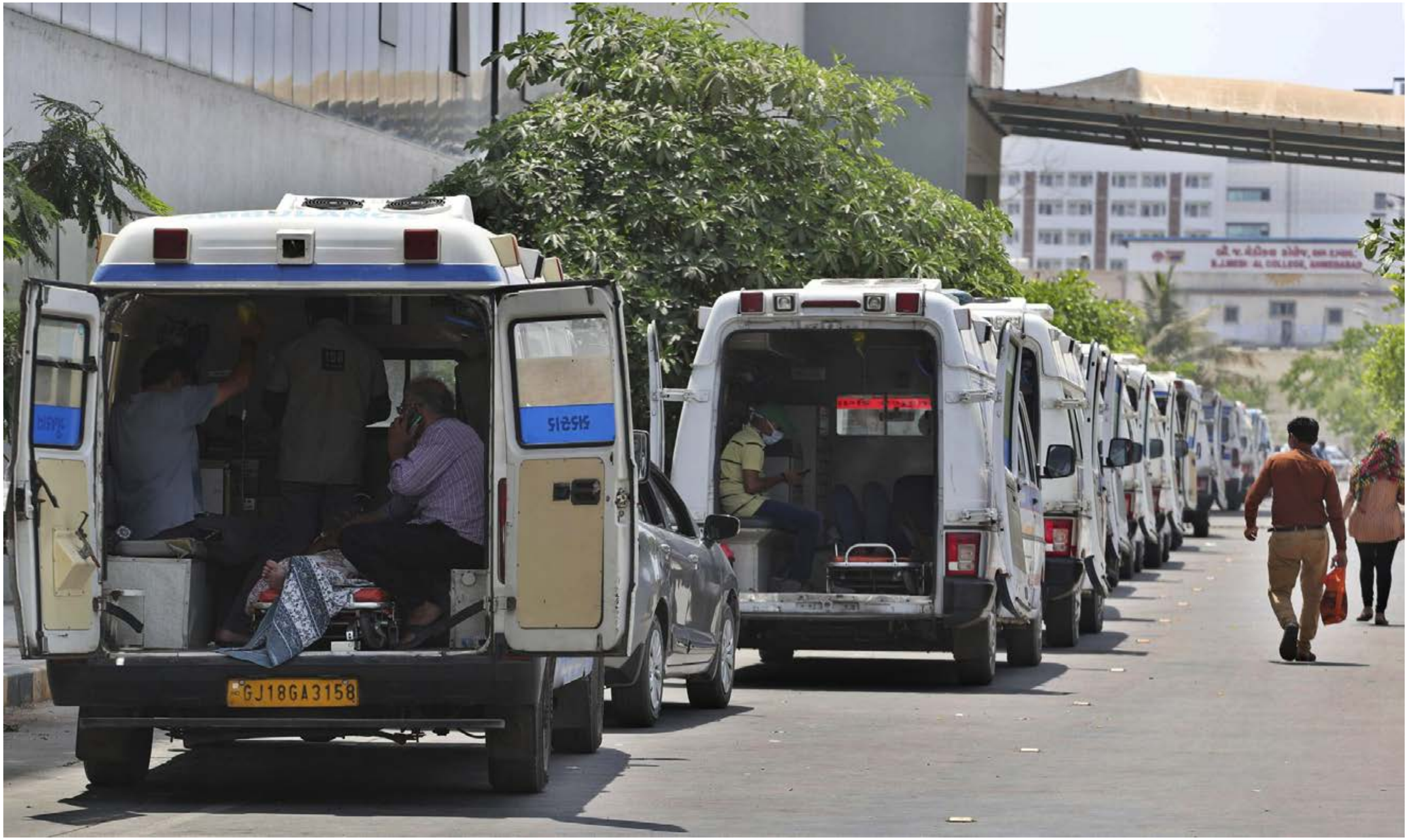


Ambulances carrying COVID-19 patients line up waiting for their turn to be attended to at a dedicated COVID-19 government hospital in Ahmedabad, Gujarat (Western part of India). Source: AP News; Dated: April 15, 2021

# People queueing for oxygen refills in Uttar Pradesh

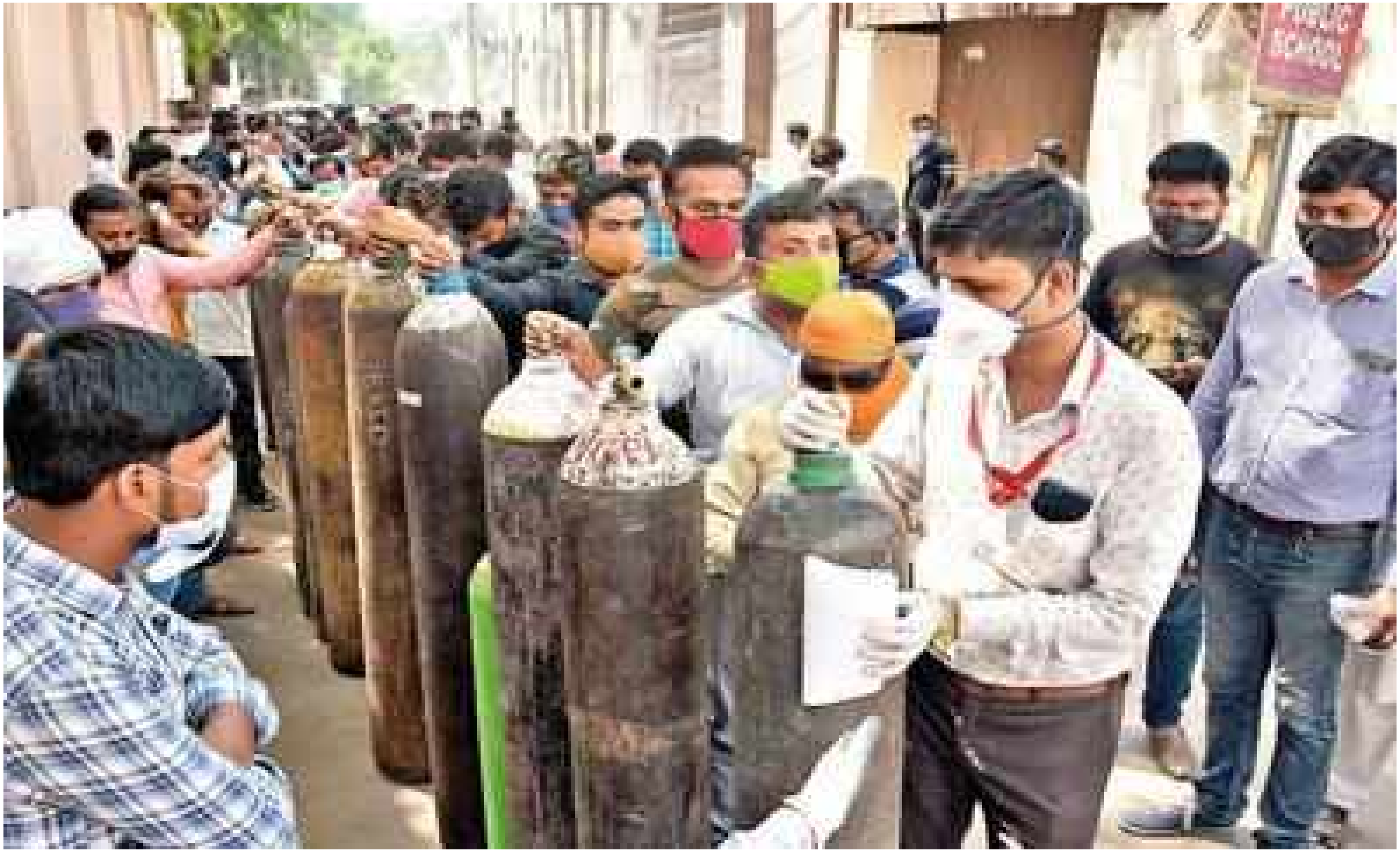

Long queue outside an oxygen plant in Talkatora, Lucknow, Uttar Pradesh (Northern part of India) to get oxygen refill.  Source: The Times of India; Dated: April 24, 2021

# People queueing for oxygen refills in Delhi

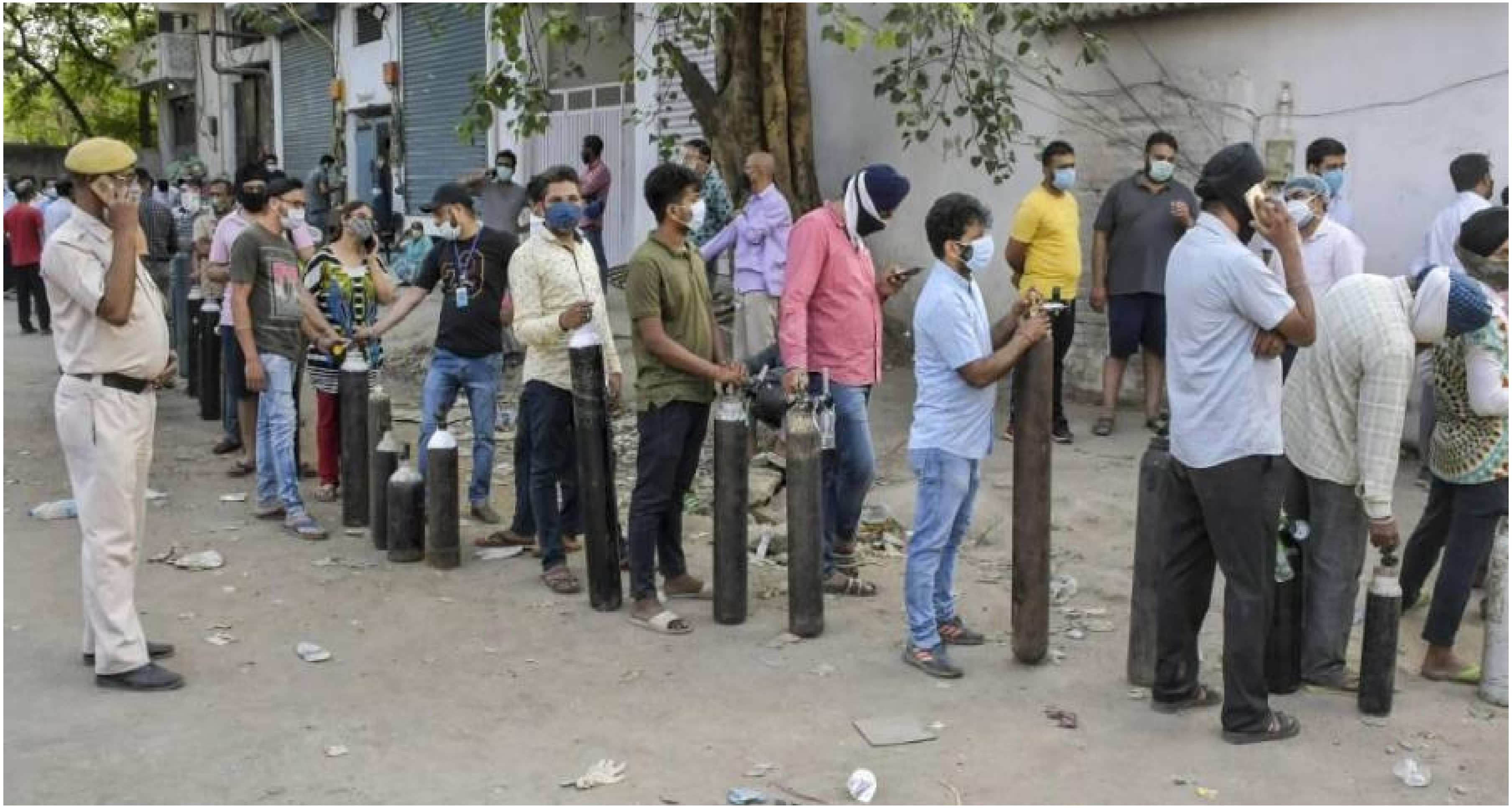

Family members of COVID-19 patients wait outside an oxygen-filling center to refill their empty cylinders as demand for oxygen rises due to spike in corona virus cases at Mayapuri in New Delhi (Northern part of India). Source: The New Indian Express; Dated: April 23, 2021

# Funeral pyres at a crematorium ground

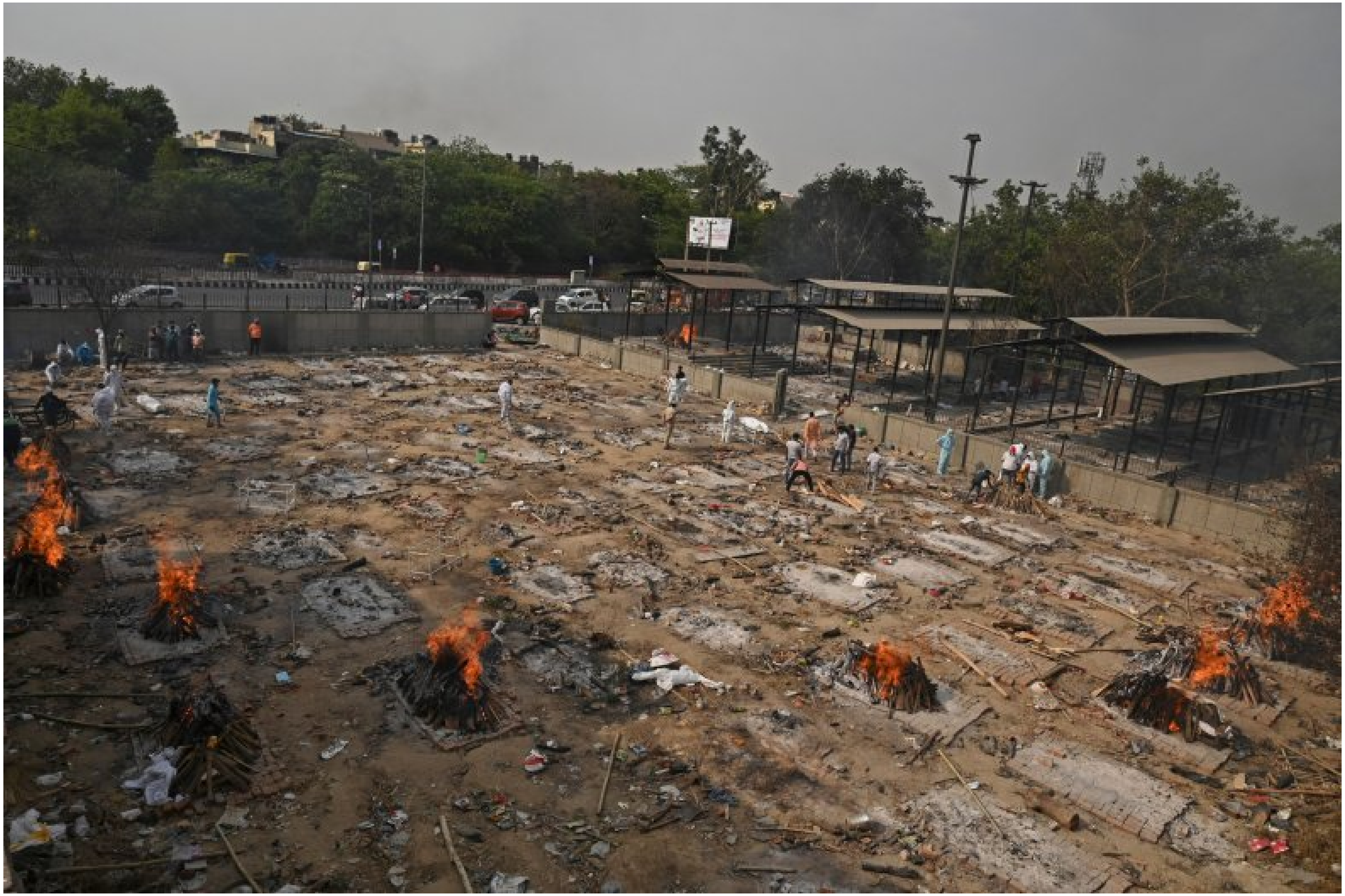

Funeral pyres of COVID-10 victims at a crematorium ground in New Delhi (Northern part of India). Source: Newsweek; Dated: May 3, 2021

# Space ran out in crematorium

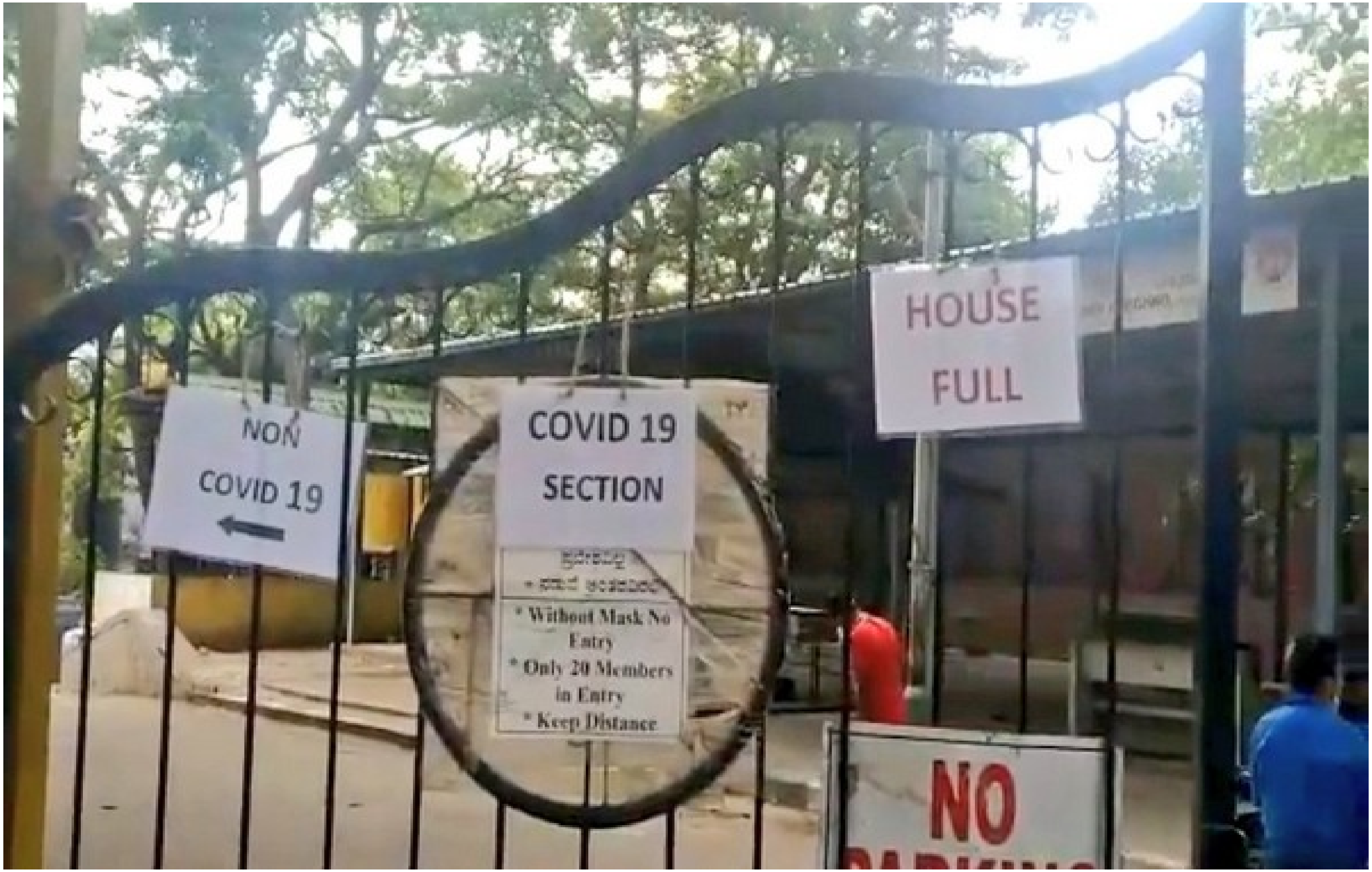


A crematorium displays a 'Housefull' board at the gate as it ran out of space in Chamrajpet, Bengaluru (Southern part of India). Source: ANI News; Dated: May 4, 2021

# Oxygen ‘langar’ organized by gurudwara

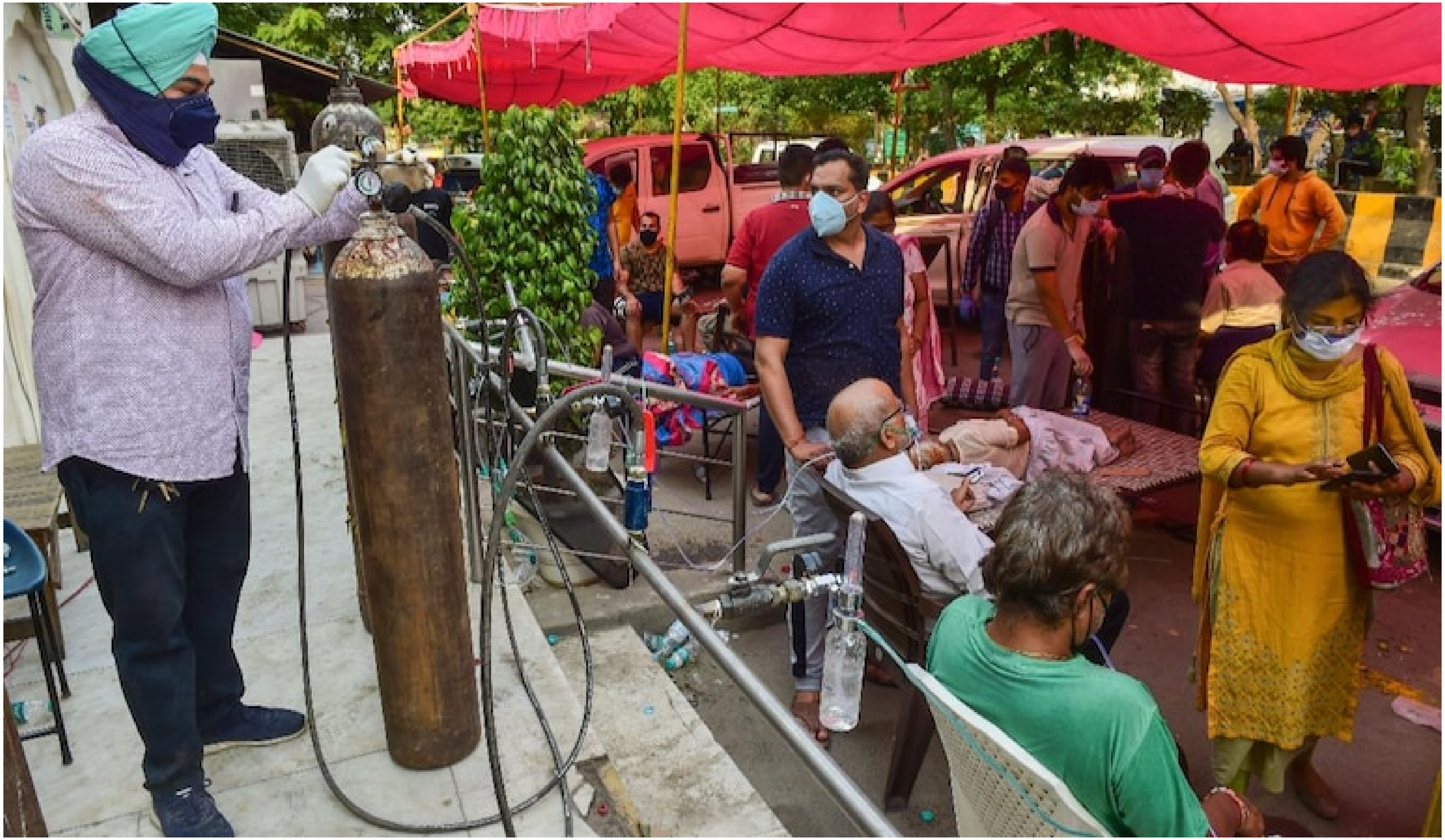

A gurudwara organized an oxygen ‘langar’ to provide relief for COVID-19 patients in Greater Kailash, South Delhi (Northern part of India). Source: India Today; Dated: April 30, 2021

# Sold SUV to donate oxygen cylinders

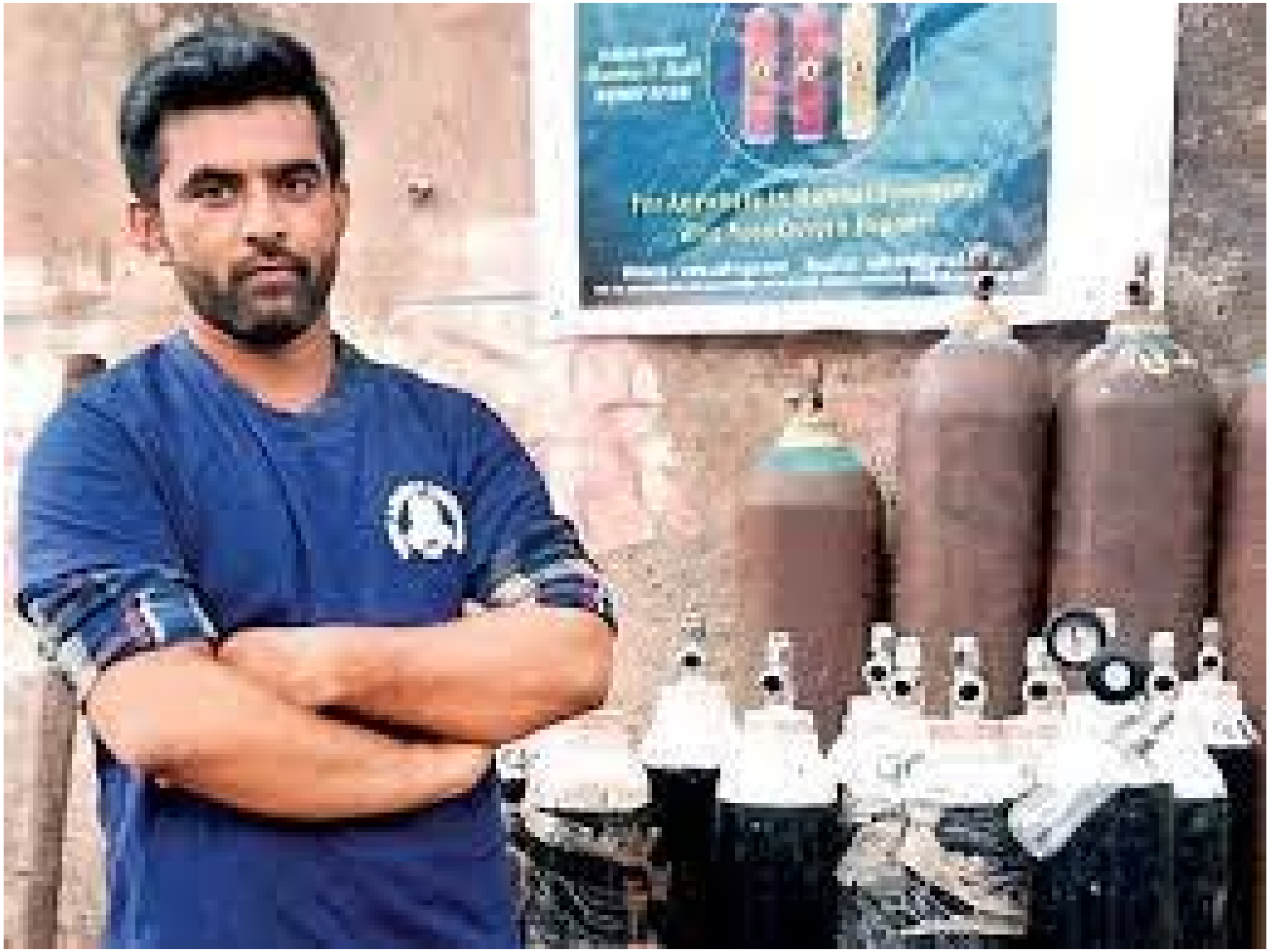

Shahnawaz Shaikh sold his Ford Endeavour to buy oxygen cylinders to 250 families for free. Source: Mumbai Mirror; Dated: June 23, 2020

# Auto-rickshaw turned into an ambulance

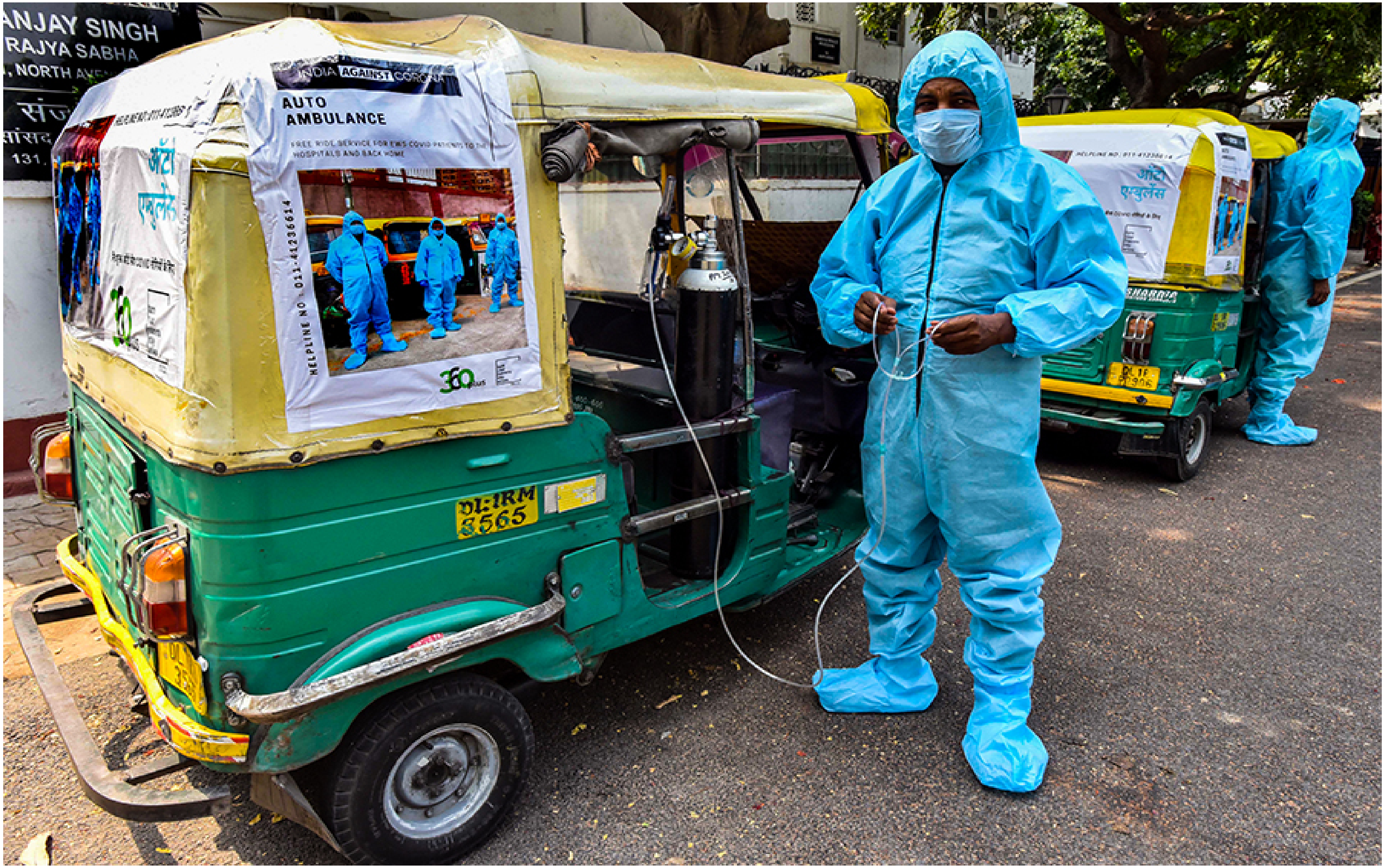


Auto-rickshaw driver in personal cover protective equipment (PPE) stands next to an autorickshaw turned into an ambulance, with oxygen facility, and offers free rides to Covid-19 patients in Delhi. Source: Forbes India; Dated: May 6, 2021

# People sharing information on oxygen concentrator

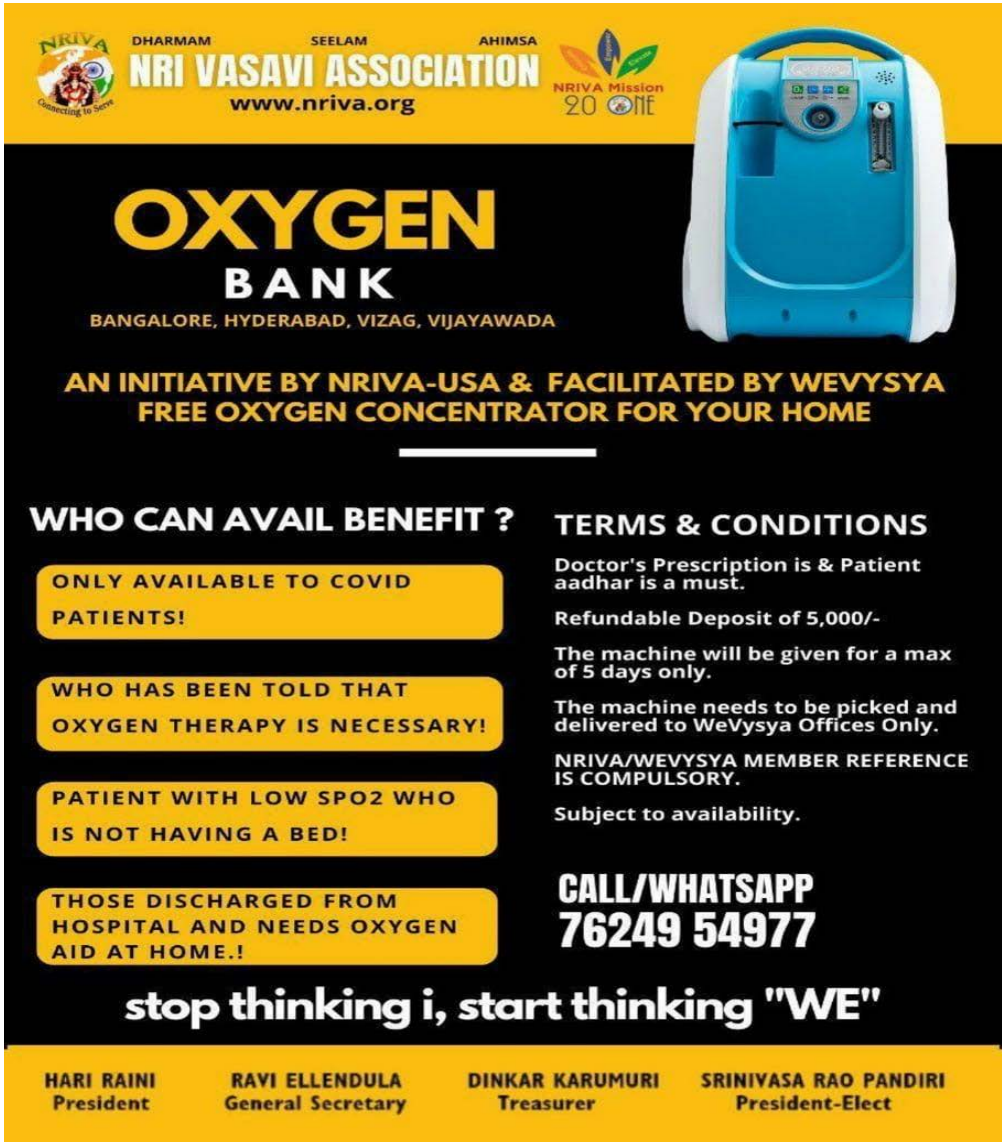


Oxygen concentrator provided by Non-Resident Indians. Source: Message shared by WhatsApp group

# CovRelief App to Help COVID-19 patients

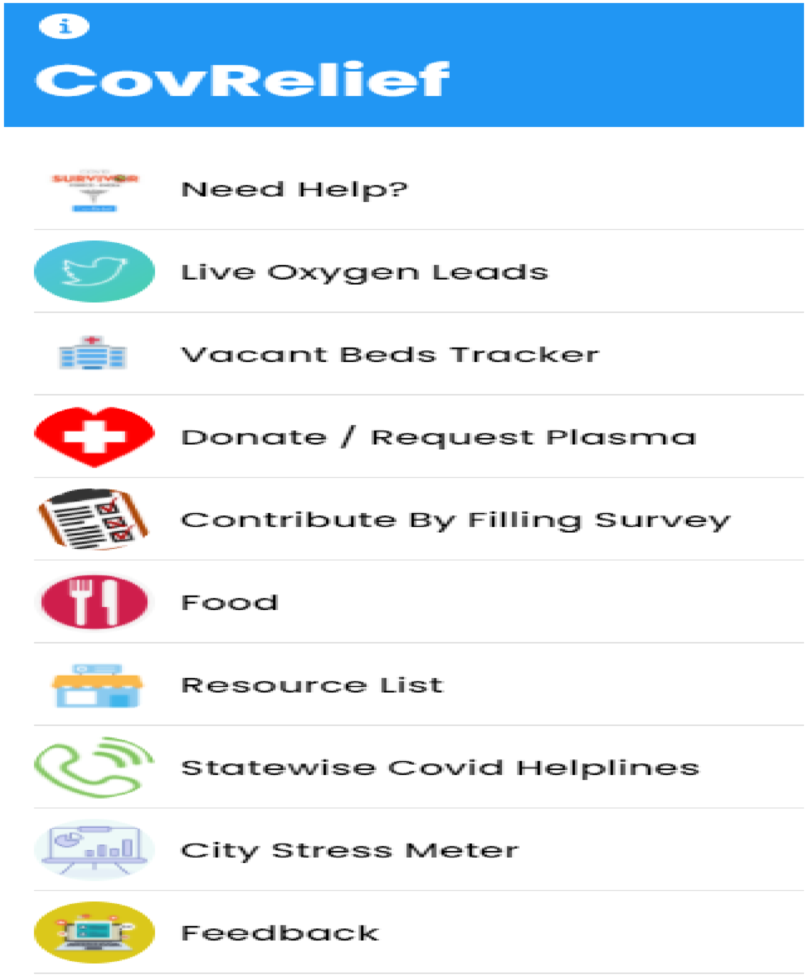


Three graduates of IIT Delhi developed a webapp CovRelief to help tracks in real-time the availability of hospital beds, lists oxygen suppliers, shares videos from doctors and has an updated list of state helpline numbers. Source: The Wire; Dated: May 7, 2021
